# Correlation analysis of larger side bone cement volume/vertebral body volume ratio with adjacent vertebral compression fractures during vertebroplasty

**DOI:** 10.3389/fendo.2023.1072087

**Published:** 2023-03-23

**Authors:** Chengqiang Zhou, Shaolong Huang, Yifeng Liao, Han Chen, Yazhong Zhang, Hua Li, Ziqiang Zhu, Yunqing Wang

**Affiliations:** ^1^ Department of Orthopedics, The Second Affiliated Hospital of Xuzhou Medical University, Xuzhou, Jiangsu, China; ^2^ Graduate School of Xuzhou Medical University, Xuzhou, Jiangsu, China

**Keywords:** osteoporotic vertebral compression fracture, bone cement volume, percutaneous vertebroplasty, percutaneous kyphoplasty, adjacent vertebral compression fracture, vertebral body volume

## Abstract

**Objective:**

To investigate the correlation analysis of larger side bone cement volume/vertebral body volume ratio (LSBCV/VBV%) with adjacent vertebral compression fracture (AVCF) in percutaneous vertebroplasty (PVP) for osteoporotic vertebral compression fracture (OVCF).

**Methods:**

A retrospective analysis of 245 OVCF patients who underwent PVP treatment from February 2017 to February 2021, including 85 males and 160 females. The age ranged from 60 to 92 years, with a mean of (70.72 ± 7.03) years. According to whether AVCF occurred after surgery, they were divided into 38 cases in the AVCF group (fracture group) and 207 cases in the no AVCF group (non-fracture group). The correlation between gender, age, bone mineral density (BMD), body mass index (BMI), thoracolumbar segment fracture, bone cement disc leakage, LSBCV, bone cement volume (BCV), VBV, LSBCV/VBV ratio (LSBCV/VBV%), and BCV/VBV% and AVCF were analyzed in both groups. Risk factors for AVCF after PVP were analyzed by multifactorial logistic regression, and then the receiver operating characteristic curves (ROC curves) were plotted to identify the critical value of LSBCV/VBV%.

**Results:**

38 patients (15.5%) developed AVCF postoperatively. Univariate analysis showed that BMD, bone cement disc leakage, LSBCV, and LSBCV/VBV% were risk factors for AVCF after PVP (P<0.05), while gender, age, BMI, thoracolumbar segment fracture, BCV, VBV, and BCV/VBV% were not significantly different in both groups (P>0.05). Multifactorial logistic regression analysis revealed that BMD, bone cement disc leakage, and LSBCV/VBV% were independent risk factors for AVCF after PVP (P<0.05). According to the ROC curve, the LSBCV/VBV% had an area under the curve of 71.6%, a sensitivity and specificity of 89.5% and 51.7%, respectively, and a critical value of 13.82%.

**Conclusion:**

BMD, bone cement disc leakage and LSBCV/VBV% are independent risk factors for AVCF after PVP. With LSBCV/VBV at 13.82%, the incidence of AVCF significantly increased.

## 1 Introduction

As populations age, the incidence of osteoporotic vertebral compression fractures (OVCFs) increases, accompanied by acute and chronic pain and progressive spinal deformities that decrease the quality of life and increase mortality (1). Therefore, attention must be drawn to developing better treatments for OVCF. Percutaneous vertebroplasty (PVP) or percutaneous kyphoplasty (PKP) is one of the effective and widely accepted methods for treating OVCF and is done by inserting cement into the fractured vertebrae for fixation to relieve pain and prevent further collapse of the vertebral body. However, some patients develop adjacent vertebral compression fractures (AVCF) within some time after PVP, which affects treatment outcomes and the quality of patient survival. Studies suggest that there are many reasons for developing AVCF after PVP (2–4). However, the amount of bone cement and its distribution pattern that alters the surgical vertebral stiffness is considered to be one of the main causes (5, 6).

Current studies on AVCF development after PVP mainly focuses on the puncture approach, bone cement volume (BCV), and BCV/vertebral body volume ratio (BCV/VBV%). In PVP, the vertebral body is unevenly filled with bone cement, often with excess on one side over the other, which leads to the uneven elastic modulus of the neighboring vertebrae, and ultimately into AVCF. Fewer systematic reports correlate larger side bone cement volume (LSBCV), LSBCV/VBV% ratio, and AVCF. Therefore, our present study focuses on the factors that influence AVCF and its correlation with LSBCV/VBV%.

## 2 Materials and methods

### 2.1 General information

**Figure 1:**
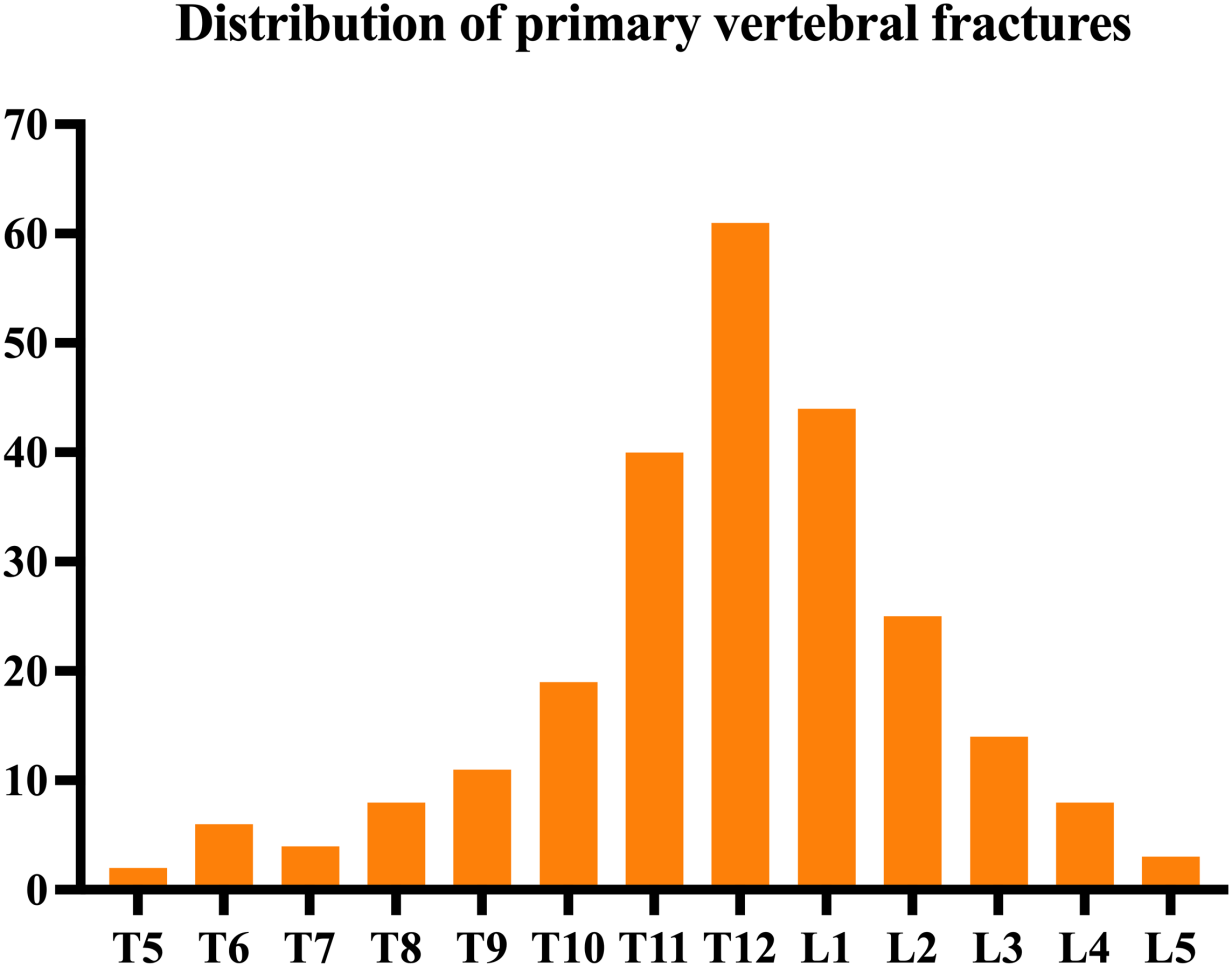
Distribution of primary vertebral fractures.

Between February 2017 and February 2021, 245 patients (male: 85; female: 160) underwent PVP for OVCF in our hospital. The average age was 70.72 ± 7.03, with a range of 60-92 years. The patients reported fracture segments: T5, two cases; T6, six cases; T7, four cases; T8, eight cases; T9, 11 cases; T10, 19 cases; T11, 40 cases; T12, 61 cases; L1, 44 cases; L2, 25 cases; L3, 14 cases; L4, eight cases; and L5, three cases (**Figure 1**). Inclusion criteria: (1) single-segment OVCF patients with obvious low back pain; (2) bone mineral density (BMD) with T-value ≤ -2.5; (3) PVP operation; (4) bilateral puncture; (5) the compression ratio of the injured spine was ≤ 1/3, T2W1 of the injured spine was a high signal on MRI, and edema signal was present on fat-suppressed sequence imaging; (6) posterior wall of damaged vertebrae was integrated, and the spinal canal was not compressed with no signs and symptoms of nerve compression upon physical examination; and (7) have complete clinical, imaging and follow-up data. Exclusion criteria: (1) infection- or tumor-related pathological fractures; (2) severe cardiac, pulmonary, hepatic, or renal insufficiency; (3) cannot tolerate surgery in the prone position; (4) coagulation disorders; (5) less than 1 year of follow-up. The average follow-up time of all patients was (17.09 ± 3.43) months. Two groups were divided according to whether AVCF occurred after surgery: 38 cases in the group with AVCF (fracture group) and 207 cases in the group without AVCF (no fracture group).

### 2.2 Surgical method

Intravenous access was established before surgery and necessary vital signs were monitored. The patient was placed prone on the operating bed, with both hands lifted and cushions to support their shoulders and pelvis so the spine is extended backward to restore the injured vertebrae. After the G-arm X-ray machine was positioned, the projection of the injured vertebra on the body surface was marked, routine disinfection after towel laying was done, and the operation was performed under local anesthesia. According to the puncture site of the thoracic and lumbar vertebrae, a puncture with a diameter matching that of surgical instruments was selected. The lower thoracic and lumbar spine can be punctured using a needle with a diameter of 3.0 mm. A bilateral pedicle approach is used for puncture under the monitoring of G-arm fluoroscopy. The 4.2 mm operating sleeve was changed after a successful puncture. The anterior middle 1/3 of the vertebra was drilled with a vertebral body drill, and a cement pusher was inserted to slowly push the cement under X-ray fluoroscopy, no more than 0.3 mL each time and the doctors observed any bone cement leakage, and recorded the total amount of bone cement, and the average amount of bone cement in the thoracic spine was 2.5-4 ml and 3.5-5 ml in the lumbar spine. All patients used the same puncture point, puncture angle and injection speed during the operation. Patients were allowed to wear waist circumference for bedside activities 1 day after surgery and were discharged from the hospital with standardized anti-osteoporosis treatment.

### 2.3 Image evaluation

A 3D *computerized tomography* (CT) reconstruction was performed on the patient 3 days after surgery, and the CT image data were exported as DICOM format files, which were then imported into Mimics 21.0 (Materiallisesoftwar, Belgium) software. All VBV, BCV, and LSBCV were measured, respectively, and LSBCV/VBV% and BCV/VBV% were calculated.

**Figure 2:**
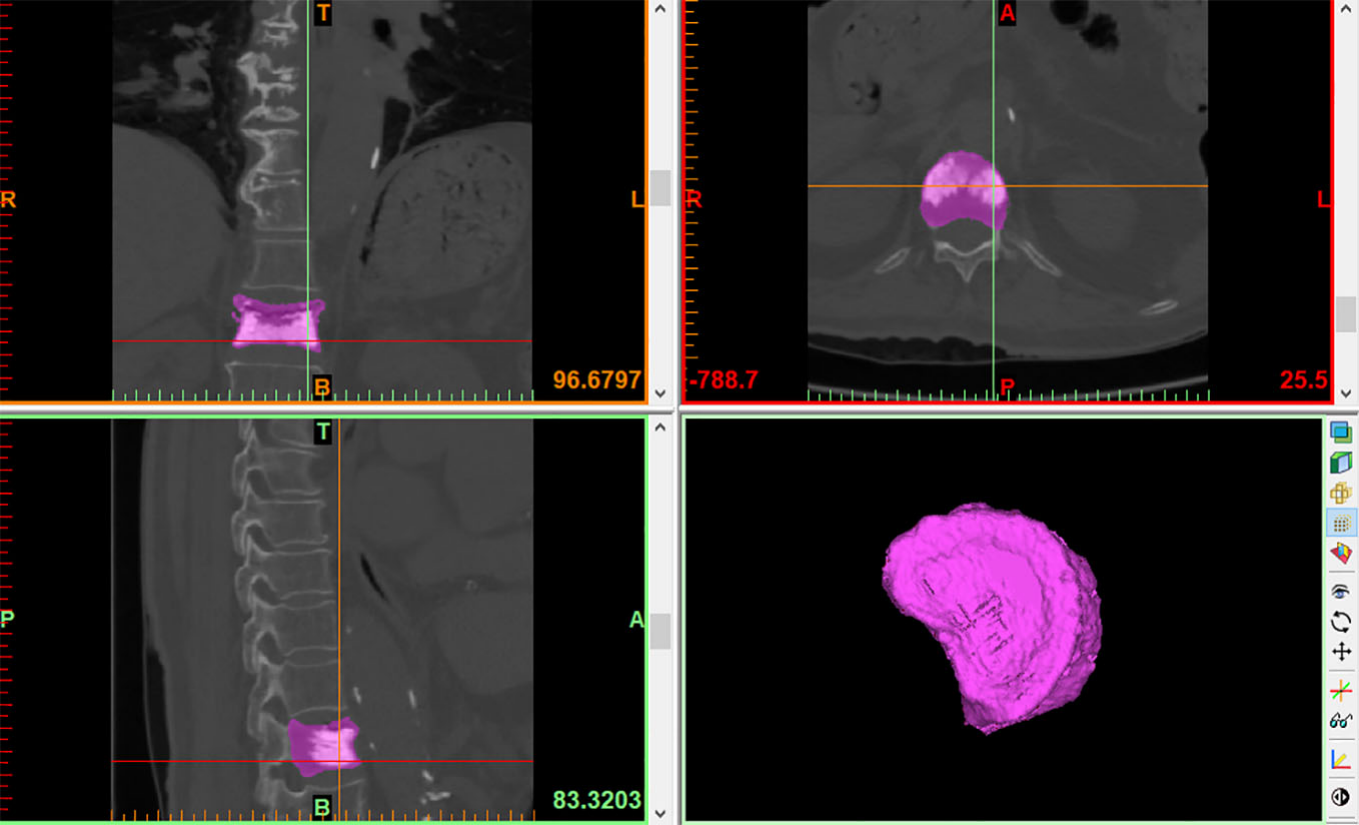
VBV.

VBV: Use the threshold segmentation tool to quickly separate the bone threshold (226-3071Hu), extract bone tissue, and perform image segmentation. After positioning the surgical vertebral body, the bilateral transverse processes, pedicles, and laminae were erased using the edit mask function, and the gap was repaired using the gap repair function. Finally, the vertebral was reconstructed by the 3D reconstruction function, and the VBV was calculated (**Figure 2**).

**Figure 3:**
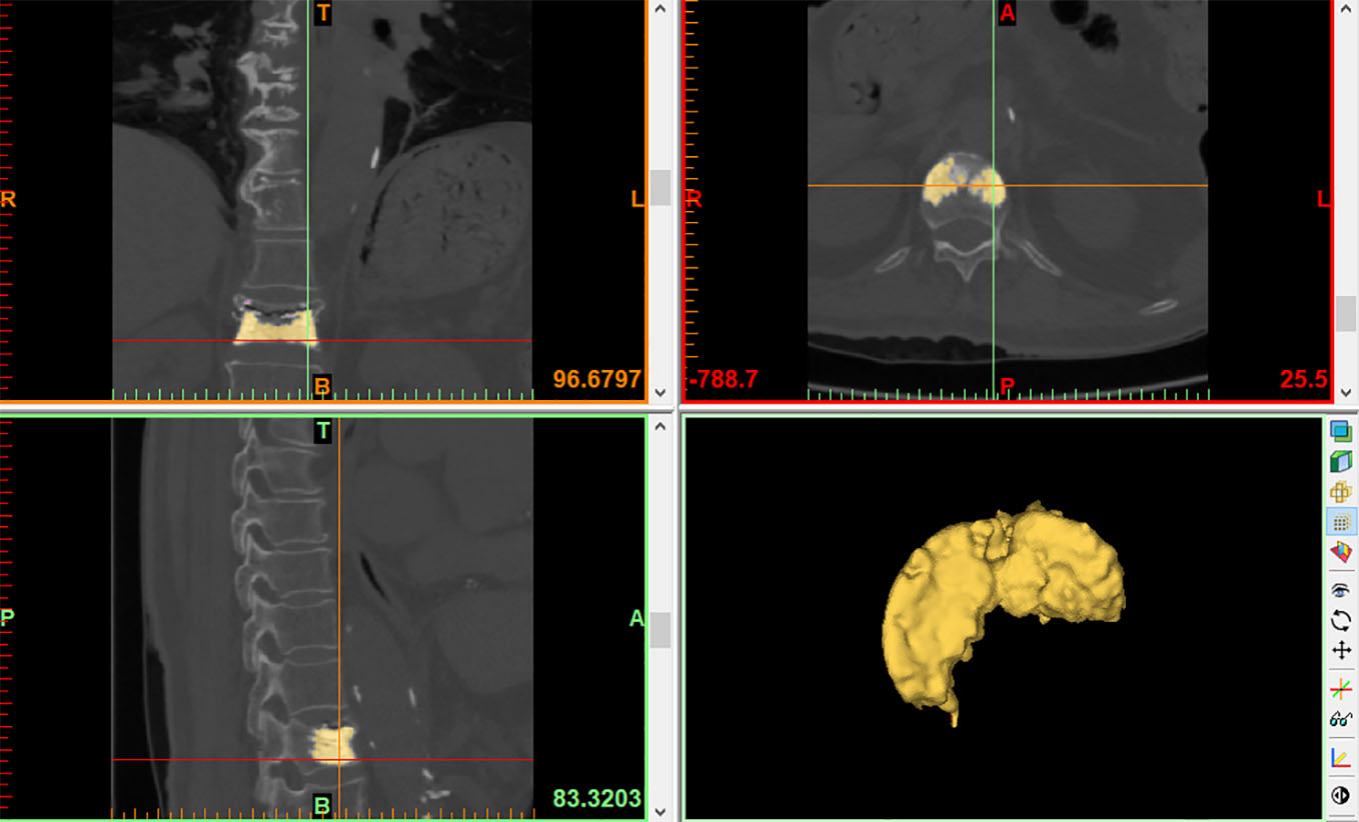
BCV.

BCV: Since the density of bone and bone cement are different, the threshold is adjusted to the bone cement threshold (1000-3071Hu) and bone cement is extracted. After the software automatically outlines the bone cement boundary, the edit mask function is used to erase the part of bone cement that leaks outside the vertebral body. Finally, bone cement reconstruction was performed by the 3D reconstruction function and BCV was calculated (**Figure 3**).

**Figure 4:**
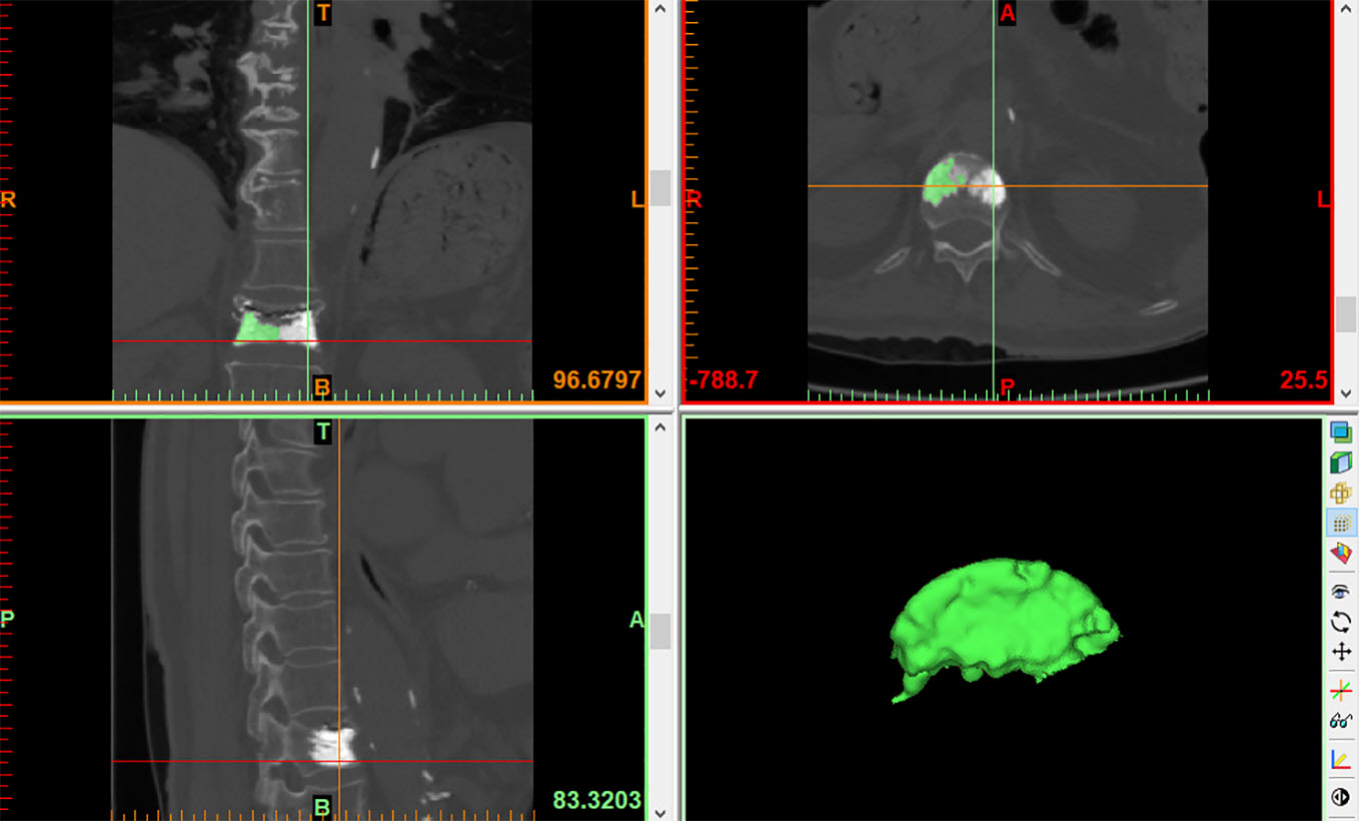
LSBCV.

LSBCV: The LSBCV is obtained by dividing the vertebral body coronally into two equal parts on the left and right and the edit mask function was used to erase the side with a smaller volume of bone cement. The LSBCV was then calculated by the reconstruction function (**Figure 4**).

### 2.4 Observation indicators

The gender, age, body mass index (BMI), BMD, thoracolumbar fracture segments, bone cement disc leakage, and LSBCV, BCV, VBV, LSBCV/VBV%, BCV/VBV% indicators that were observed in the fracture and non-fracture group.

### 2.5 Statistical analysis

We used the SPSS 26.0 software (IBM, Armonk, NY, USA) to analyze the data. The measurement data were expressed as mean ± standard deviation, and independent sample t-tests were used for comparison between groups. The count data were analyzed by the chi-square test. Indicators were screened by univariate analysis first, and those indicators with statistically significant differences were then subjected to multifactorial logistic regression analyses. We also constructed the ROC curve to calculate the area under the curve as well as the critical value of LSBCV/VBV%.

## 3 Results

All patients completed the surgery successfully. The length of the operation was between 33-62 minutes (average: 42.49 ± 5.06). A total of 245 patients received final follow-up, of which 38 (15.51%) patients developed AVCF and in 20 (8.16%) patients, it occurred within 6 months after the first operation and 12 (4.90%) patients reported AVCF within 1 year after their first operation.

Univariate analysis shows that gender, age, BMI, thoracolumbar fracture, BCV, VBV, and BCV/VBV% did not affect AVCF after PVP surgery (P > 0.05). The BMD T-value, bone cement disc leakage, LSBCV, and LSBCV/VBV% significantly affected the AVCF after PVP (P < 0.05) (**Table 1**).

**Table 1:** Univariate analysis that affects AVCF.

Relevant factors	Fracture group (n=38)	Non-fracture group (n=207)	*t/x^2^*	*P*
Gender (M/F)	13/25	72/135	0.005	0.946
Age	70.18 ± 8.27	69.96 ± 7.78	0.164	0.870
BMI	23.17 ± 2.74	23.02 ± 3.32	0.267	0.790
BMD (T)	-3.40 ± 0.56	-3.14 ± 0.59	-2.494	0.013
Thoracolumbar fracture (T_10_~L_2_)	32/38	157/207	1.274	0.259
Bone cement disc leakage	6/32	11/196	3.954	0.047
LSBCV	4.27 ± 0.90	3.72 ± 1.00	3.134	0.002
BCV	6.37 ± 1.37	6.27 ± 1.31	0.429	0.668
VBV	25.63 ± 5.18	26.61 ± 5.80	-0.978	0.329
LSBCV/VBV%	16.90 ± 3.00	14.14 ± 3.18	5.158	<0.001
BCV/VBV%	25.45 ± 5.92	24.08 ± 5.48	1.400	0.163

BMI, body mass index; BMD, bone mineral density; LSBCV, larger side bone cement volume; BCV, bone cement volume; VBV, vertebral body volume; LSBCV/VBV%, larger side bone cement volume/vertebral body volume ratio; BCV/VBV%, bone cement volume/vertebral body volume ratio.

Multifactorial logistic regression analysis showed that low BMD, bone cement disc leakage, and LSBCV/VBV% were risk factors for AVCF development after PVP surgery (P < 0.05) (**Table 2**).

**Table 2:** Multifactorial logistic regression analysis that affects AVCF.

Influencing factors	B	S.E	Wald	*P*	OR	95% CI
BMD	-0.780	0.318	6.042	0.014	0.458	0.246~0.854
Bone cement disc leakage	1.353	0.604	5.020	0.025	3.869	1.185~12.637
LSBCV	0.085	0.236	0.130	0.719	1.089	0.686~1.728
LSBCV/VBV%	0.254	0.076	11.289	0.001	1.289	1.112~1.496

BMD, bone mineral density; LSBCV, larger side bone cement volume; LSBCV/VBV%, larger side bone cement volume/vertebral body volume ratio.

**Figure 5:**
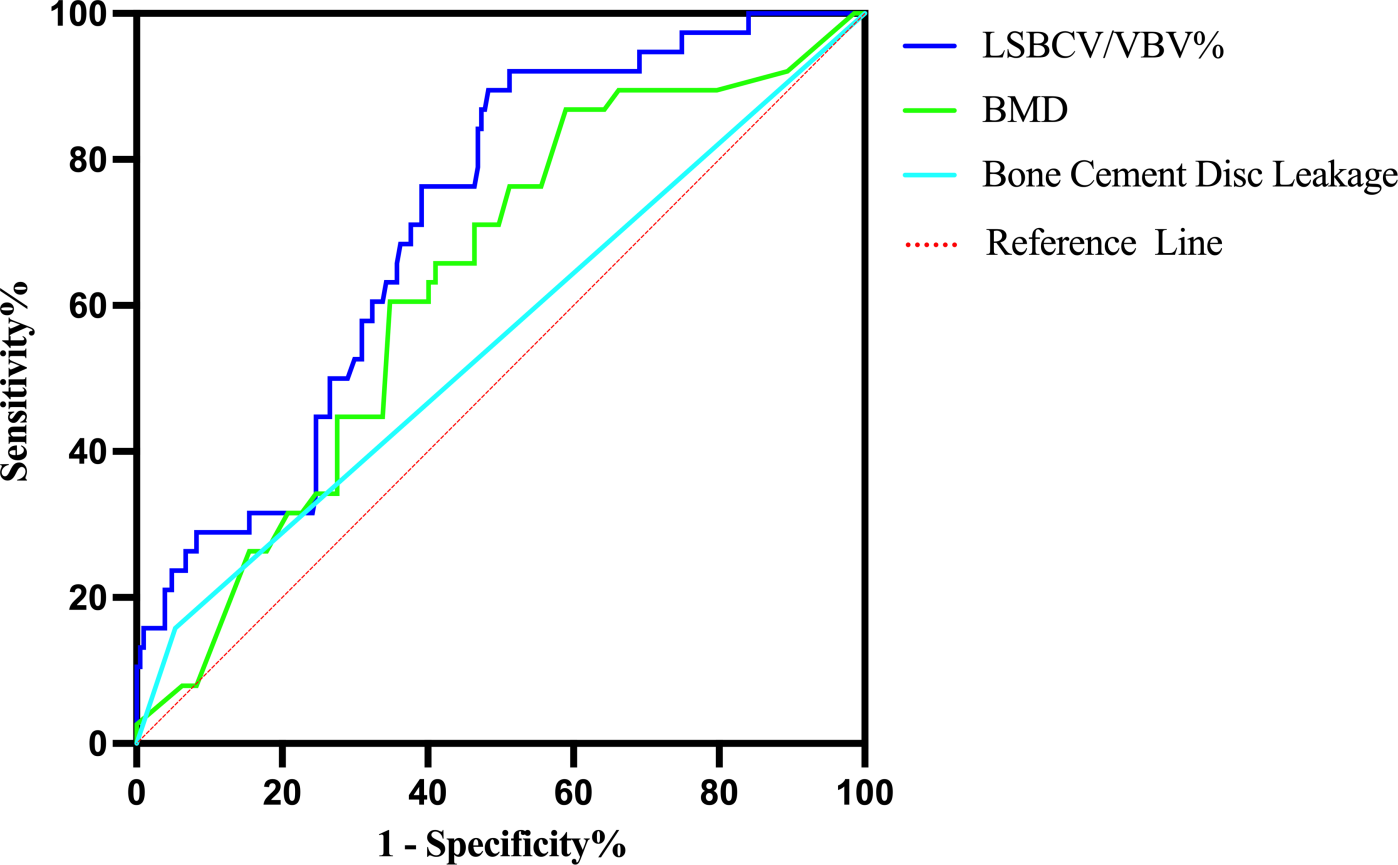
ROC curve for the diagnosis of AVCF.

ROC curves of BMD, bone cement disc leakage and LSBCV/VBV% were constructed (**Figure 5**). The area under the curve was 63.1% for BMD, 55.2% for bone cement disc leakage, and 71.6% for LSBCV/VBV% (**Table 3**).

**Table 3:** Area under the ROC curve for the diagnosis of AVCF.

Factors	AUC	*P*	95% CI	
			Lower	Upper
BMD	0.631	0.010	0.540	0.722
Bone cement disc leakage	0.552	0.305	0.447	0.657
LSBCV/VBV%	0.716	<0.001	0.637	0.794

BMD, bone mineral density; LSBCV/VBV%, larger side bone cement volume/vertebral body volume ratio.

**Figure 6:**
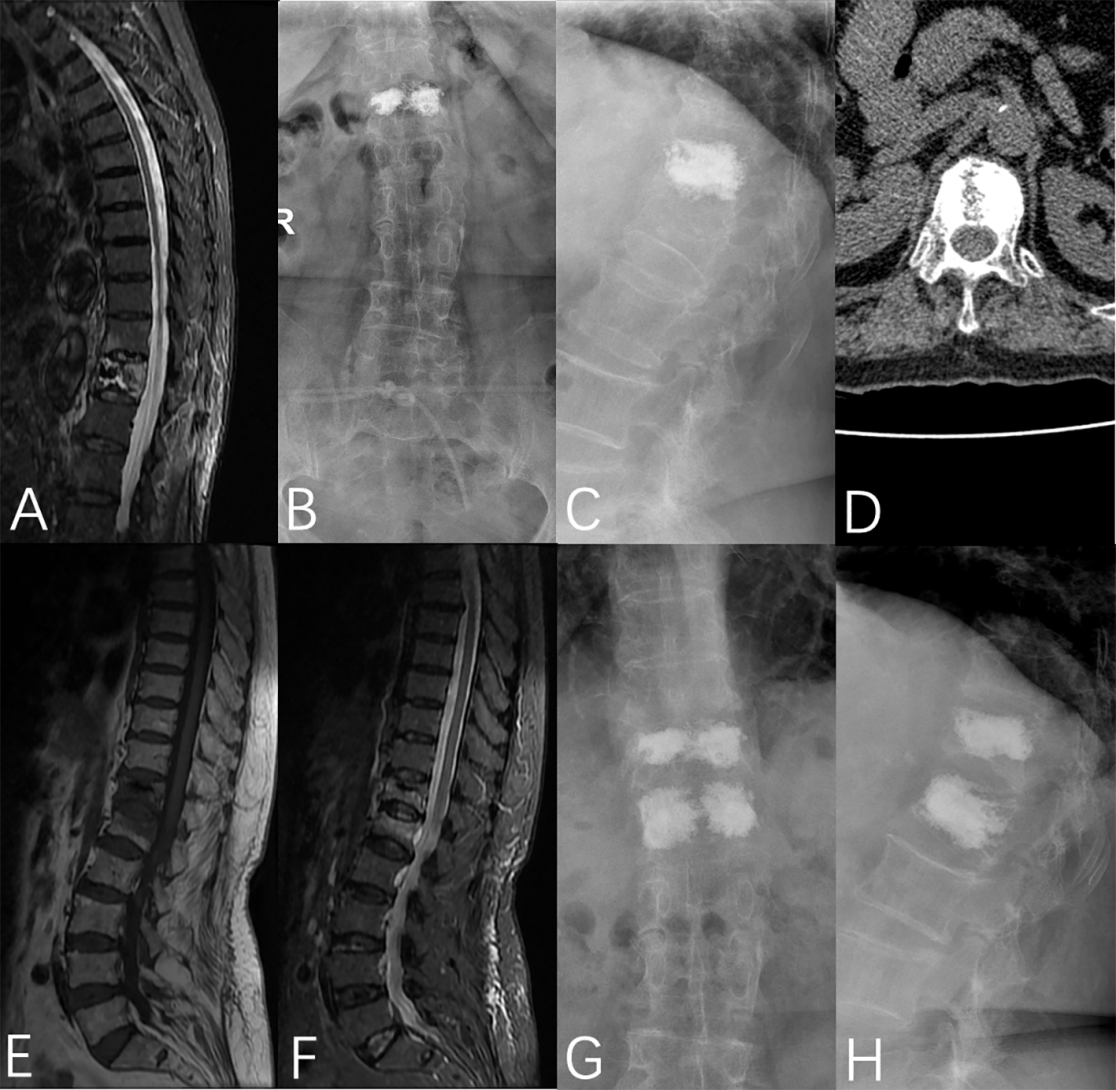
A 76-year-old female was admitted to the hospital with no obvious cause of low back pain for 1 week, was diagnosed with OVCF (T12), and underwent PVP under local anesthesia on the third day after admission. **(A)** Preoperative fat-suppressed image showed T12 vertebral fracture, **(B, C)** T12 vertebral bone cement filling could be seen in the anterior and lateral X-rays after the operation, **(D)** Postoperative CT showed that the bone cement was unevenly distributed on the bilateral sides of the vertebrae, **(E, F)** MRI showed L1 vertebral fracture 5 months after the operation, **(G, H)** T12 and L1 vertebral bodies were filled with bone cement in the anterior and lateral X-rays after the operation.

The sensitivity and specificity of LSBCV/VBV% corresponded to 89.5% and 51.7%, respectively, and the cut-off value at this time was 13.82% (**Table 4**). Typical cases are shown in **Figure 6**.

**Table 4:** Sensitivity and specificity corresponding to LSBCV/VBV%.

Factor	Sensitivity	Specificity
LSBCV/VBV% (13.82)	0.895	0.517

LSBCV/VBV%, larger side bone cement volume/vertebral body volume ratio.

## 4 Discussion

At present, PVP is a common, minimally invasive technique for treating OVCF that can effectively relieve low back pain and promote early functional activity (7). However, patients are usually subjected to the reoccurrence of surgical/non-surgical vertebral fractures after surgery. A meta-analysis showed (8) that the incidence of refracture after PVP varied from 3.21% to 63%. YI et al. (9) showed that the incidence of AVCF in patients with OVCF and PVP and PKP was 19.59% at 1-year post-surgery. In addition, MAZZANTINI et al. (10) showed a 27.8% incidence of AVCF in patients with vertebral body strengthening at 39 months postoperative follow-up. The incidence of AVCF during patient follow-up was 15.5% in this study and was consistent with the above literature. Moreover, there is no unified view of the risk factors of AVCF after vertebroplasty. Several factors can influence its occurrence, as shown in previous studies (11, 12). In this study, our univariate analysis found that low BMD, bone cement disc leakage, LSBCV, and LSBCV/VBV% were risk factors for AVCF development after PVP. A through multivariate analysis showed that BMD, bone cement disc leakage and LSBCV/VBV% were independent risk factors for AVCF after vertebral augmentation.

Several studies have shown that BMD can reflect the degree of osteoporosis (13–15). The lower the BMD, the more serious the degree of osteoporosis, and vertebral body fracture can be caused by a slight external force. Rho et al. (16) found that a decrease in BMD and leakage of bone cement into the intervertebral disc also were influential factors in AVCF. Lu et al. (17) conducted a retrospective study of 204 patients after vertebroplasty, and found that lower BMD T values were a risk factor for AVCF after vertebroplasty. Similarly, in this study, low BMD was also found to be a risk factor for AVCF after vertebroplasty by univariate analysis and binary logistic regression analysis, and the lower the BMD T value, the greater the risk for AVCF. PVP can be treated by fixing the fractured vertebral body through bone cement, and this has no therapeutic effect on osteoporosis. However, there still lies a risk of fracture in other vertebral bodies after PVP, especially the adjacent vertebral bodies due to interference of vertebral bodies with high elastic modulus after enhancement. Thus, for patients with low BMD, anti-osteoporosis therapy should be actively performed after PVP, and regular imaging examinations should also be carried out to prevent the occurrence of postoperative AVCF.

This study shows that the leakage of bone cement can also be a risk factor in AVCF development. Bone cement leakage is a common complication after PVP and PKP, with its incidence ranging from 11% to 73% (18). After the occurrence of a vertebral compression fracture, the internal bone trabeculae become dense due to compression and a hematoma is often present near the fracture line, which requires an increase in pushing pressure of the bone cement to diffuse the cement in the fracture gap, thus predisposing to a cement leakage (19). The bone cement leaks into the intervertebral disc, causing a transient fever, changing the physicochemical properties of the discs and destroying its structure, accelerating disc degeneration, and making it lose its buffering effect, thus leading to abnormal load conduction. At the same time, this leak can also increase the stress of adjacent vertebral bodies due to the “pillar effect” that increases the risk of AVCF. A meta-analysis showed that cement leakage was a risk factor for AVCF after PVP in patients with OVCF, while cement volume was not a risk factor for AVCF (2), and the results are consistent with the results of our study.

In vertebroplasty, the cement is usually unevenly distributed on both sides of the vertebrae. We believe that this may be related to the angle of bilateral puncture and the different pressure and speed of bilateral push injection during bilateral puncture, often resulting in more bone cement on one side than on the other. This causes the vertical compression force of the entire vertebral body to shift to the other side, which increases the vertical stress in the neighboring vertebrae. However, after the vertebral body is hardened by bone cement, the stiffness is too large, and the stress distribution is uneven that then transfers to the neighboring vertebrae and discs (20), resulting in AVCF. In this study, we maintained the same injection point, angle of puncture, speed of pushing, and amount of bone cement on both sides during bilateral punctures in all patients to ensure that the proportion of bone cement on both sides was as equal as possible. An OVCF model was developed to compare the stiffness of the entire vertebral body and both sides of the vertebral body after unilateral and bilateral percutaneous vertebroplasty. It was found that when the bone cement was confined only to the punctured side, the unreinforced side was less safe than the reinforced side, and when bone cement distribution extended to the midline and filled the non-punctured side, a balance in stress was obtained on both sides of the vertebral body (21). At the same time, in vertebroplasty, the likelihood of cement leakage increases when it is overly concentrated on one side of the vertebral body compared to an even distribution on both sides. However, there lacks relevant literature which systematically studies the correlation of LSBCV and LSBCV/VBV% with AVCF. Previous studies have mostly concentrated on the impact of BCV and BCV/VBV% on vertebroplasty. Jin et al. (22) concluded that to ensure surgical efficacy and reduce complications, BCV/VBV% should be at least 11.64%. However, in these studies, BCV and BCV/VBV% were mostly described by the amount of cement injected and by a CT scan. The BCV refers to the distribution of bone cement in the vertebral body along the trabecular bone or the gap between the fracture line that forms a 3D spatial structure composed of bone cement, trabecular bone, and its gap. However, during the puncture, there will be some bone cement remaining in the puncture cannula as well as leakage of bone cement outside the vertebral body, which results in an inconsistency between injected cement volume and the actual BCV within the vertebra. Also, the VBV calculated by CT can differ from the true VBV, so it is not possible to calculate BCV/VBV% accurately in this manner. Therefore, we imported the patient’s CT data into the Mimics software and used its 3D reconstruction function to accurately calculate BCV, VBV, and LSBCV to derive LSBCV/VBV% and BCV/VBV%, and included them in the univariate analysis and binary logistic regression analysis. We found that BCV/VBV% was not a risk factor for AVCF, whereas LSBCV/VBV% was the risk factor for AVCF. Thus, by establishing the ROC curve, we found that the optimal cut-off value for LSBCV/VBV% to diagnose AVCF was 13.82%, with a sensitivity of 89.5% and specificity of 51.7%. Therefore, when LSBCV/VBV% exceeded 13.82%, the incidence of AVCF increased significantly.

However, there are some limitations to our study. First, this study was a retrospective single-center study with a short follow-up period and a small sample size. We hope to further analyze other factors that affect AVCF by carrying out a large-sample, multicenter, prospective study in the future. Second, we only included PVP, not PKP, because PKP and PVP had different effects on the results (23). Lastly, we only studied the effects of cemented disc leakage on AVCF and did not examine if a leakage in bone cement to other sites could also affect AVCF.

In conclusion, BMD, bone cement disc leakage, and LSBCV/VBV% were independent risk factors for AVCF development after PVP. When LSBCV/VBV% reached 13.82%, the incidence of AVCF significantly increased. Therefore, to prevent the occurrence of AVCF after PVP, clinicians should keep the injection point, angle, pressure, and speed of pushing the puncture as consistent as possible during bilateral punctures. When bone cement disc leakage is found during operation, more vigilance is required, and at the same time, attention should also be paid to standardized anti-osteoporosis treatment to reduce the incidence of AVCF after PVP.

## Data availability statement

The raw data supporting the conclusions of this article will be made available by the authors without undue reservation.

## Ethics statement

The studies involving human participants were reviewed and approved by Ethics Committee of the Second Affiliated Hospital of Xuzhou Medical University. The patients/participants provided their written informed consent to participate in this study. Written informed consent was obtained from the individual(s) for the publication of any potentially identifiable images or data included in this article.

## Author contributions

The first draft of the manuscript was written by CZ. Data collection and analysis were performed by CZ, SH, YL. Interpretation of data was performed by HC, YZ, HL, ZZ and YW had substantively revised it. All authors contributed to the article and approved the submitted version.
